# On Averting Negative Emotion: Remedying the Impact of Shifting Expectations

**DOI:** 10.3389/fpsyg.2018.02121

**Published:** 2018-11-20

**Authors:** Cecile K. Cho, Theresa S. Cho

**Affiliations:** ^1^International Business Department, Korea University Business School, Seoul, South Korea; ^2^Strategy and International Management Department, College of Business Administration, Seoul National University, Seoul, South Korea

**Keywords:** emotion, expectation, motivated goals, comparison standards, disappointment, anticipatory strategies

## Abstract

This paper examines how people anticipate negative emotions when faced with an uncertain outcome and try to manage their expectations. While extant research streams remain equivocal on whether managing expectations always succeeds, this research examines situations in which setting a low expectation can have an adverse emotional impact and suggests ways to alleviate this negative consequence. Using goal setting and a false-feedback paradigm, we show that, although individuals who set low goals to manage expectations can end up feeling more disappointed than those who set high goals (study 1), this negative impact can be avoided when individuals are reminded of their initial goals at feedback, or made aware of inaccuracies in forecasting their future emotion (studies 1 and 2).

## Introduction

Anticipating the future, the capacity to represent future events flexibly and imagine diverse possible outcomes are fundamental to human being’s survival. Individuals anticipate circumstances by taking expected future consequences into consideration when setting present goals and standards (Aspinwall, 2005). The link between one’s anticipation for future outcomes and its impact on current behavior is a central topic in self-regulation (Bandura, 1982; Higgins, 1987, 1996, 1997; Carver and Scheier, 1990, 1998). Research on bracing demonstrates that people have a robust tendency to lower predictions of their own performance (Shepperd et al., 1996). In anticipation of a potentially disappointing outcome, individuals have been found to lower their expectations in order to reduce the gap between expectations and outcomes (see Carroll et al., 2006). For example, Carroll et al. (2006) found that student predictions for upcoming grades or exam scores moved from optimistic (over-estimating the actual results) to pessimistic (underestimating) as the time of receiving the grade approached (Shepperd et al., 1996). The finding from bracing research suggests that lowering expectations can indeed serve to cushion and avert disappointment when negative outcomes occur. This is because such lowered expectations are then used as a reference standard against which an outcome is judged. In this research, we investigate whether lowered expectations always serve as the reference standard for evaluating the outcome. Should the lowered expectancy fail to serve as the reference standard, the assumed cushioning effect may not take place.

Seen from a self-regulation perspective, avoiding disappointment could be conceptualized as a meta-goal that motivates the subsequent lowering of expectations. Given an uncertain future outcome, when a potential salient failure looms, a negative emotion accompanying this failure is also likely to be salient and trigger the defensive lowering of the forecasted outcome. The lay belief that one needs to “manage expectations,” as doing so would help one avoid the negative emotion of failure, is common and influences how people assess what they will find satisfactory. In this paper, we consider such lowering of expectations as synonymous with choosing an easy goal, along with implications for a self-regulation strategy whereby avoiding a negative affect is part of successful goal pursuit.

### Setting Low Goals to Manage Expectations

Goal setting is one of the most widely accepted paradigms of motivation and self-regulation behavior (e.g., Locke et al., 1990). Decades of research have consistently demonstrated that setting specific and challenging goals can powerfully motivate an individual. Recently, however, research on goals has begun to examine the negative consequences. Setting specific and challenging goals has been demonstrated to increase individuals’ engagement in unethical behavior and excessive risks, as well as to demotivate and derail goal striving (Larrick et al., 2009; Ordonez et al., 2009; Townsend and Liu, 2012). If setting a challenging goal becomes a salient reference that can derail goal striving, other research has found that setting an easy and safe goal can also be demotivating, as people compare their performance to a better imagined outcome (Cho and Johar, 2011).

In this research, we start from the premise that setting low goals demotivates and leaves one disappointed. We draw on the recent finding that evaluation standards can shift over time (Van Dijk et al., 2003; Monga and Houston, 2006; Cho and Johar, 2011). In particular, Cho and Johar (2011) found that setting goals low could be less satisfying because people often compare to a better possible outcome. In our research, we examine the conditions under which such a shifting of goal standards is likely and test ways to intervene. Across two studies, we explore conditions under which setting easy goals (even if achieved) can lead to perceptions of failure and show that reference standards used to evaluate outcomes is not the initial expectation (study 1); and, making participants aware that comparative standards can shift might remedy the perception of failure and accompanying negative emotions. In study 1, we manipulate motivational states to induce goal levels while measuring the chronic disposition to anticipate failure and negative emotions in order to predict the anticipatory lowering of goals and expectations. In study 2, we test the assumption that individuals concerned with prevention goals are more likely to manage expectations. In this way, we show that increasing awareness of the futility of trying to manage a future affect could serve to intervene against this counterproductive tendency.

### Anticipated Emotion and Goal Setting

In order to demonstrate the self-regulatory nature of a goal-lowering strategy, we draw on motivation literature to create conditions under which low-vs.-high goals are set. Similarly, research on regulatory focus suggests that avoidance orientation (prevention focus) is associated with pursuing minimal goals, whereas approach orientation (promotion focus) is associated with pursuing maximal goals (Brendl and Higgins, 1996; Forster et al., 1998; Jain et al., 2006). The motive to avoid failure is associated with the anticipated negative emotion that accompanies this failure (McClelland, 1953). The tendency to set low goals is likely to be exacerbated when consumers are concerned about minimizing the negative emotion that accompanies failure (Van Dijk et al., 2003).

## Study 1

The purpose of this study is twofold. First, we seek to show that people lower their expectations of what will be satisfactory as an anticipatory coping strategy to avoid disappointment. Second, we seek to show that lowering expectations may not help to avert disappointment even if expectations are confirmed. Consistent with previous research, our prediction is that lowering expectations will lead to feeling more, not less, disappointed, because the initially set expectation is not used as a comparison standard when the performance outcome is revealed and evaluated (Cho and Johar, 2011). In this study, we use avoidance and approach motivations to operationalize and induce setting of low and high goal levels, respectively (Friedman and Forster, 2001; Zhou and Pham, 2004). Our logic in using approach and avoidance motive as antecedents of lowering expectation comes from the motivation literature which states that avoid motivation should trigger a greater sensitivity to potential failure; the greater concern with failure and negative affect, the greater tendency to lower goals (for example, Forster et al., 1998). It also follows from the bracing literature which found that when faced with potentially negative news, people lower their expectations in order to prepare for this bad new (Shepperd et al., 1996). Therefore, we predict that under avoidance motivation, participants will lower their expectation (set lower goals), whereas under approach motivation, participants will set a comparatively higher expectation (set higher goals).

If the lowered expectation is used to evaluate the outcome (which is confirmed using the false feedback paradigm) then the measure of disappointment should be minimal. If people end up comparing their outcome to imagined outcomes that are better (“it could have been better”), then even after initially lowering their expectation strategically (Shepperd et al., 1996), the assumed benefit of the lowered expectation is likely misguided and needs to be corrected.

We draw on previous research and vary the information present at the time of performance feedback to test the preceding hypothesis that potential performance is likely to be the spontaneously recruited comparison standard (Cho and Johar, 2011). In their study, Cho and Johar (2011) showed that when respondents were provided with their performance results, they seemed to compare their outcomes to the highest potential, which likely served as a spontaneous comparison standard. However, making the initially set goal salient at the time of feedback should result in no difference between the low-and-high goal conditions: that is, both groups should compare their performance to the salient goal and realize they have met the goal and should therefore be equally satisfied. In our study, we manipulate approach and avoidance motives so as to invoke fear of failure and a negative affect. We seek to show that the desire to avoid a negative affect from failure is driving the lowering of expectation and thereby imparting a negative consequence on satisfaction because the comparison standard used is not the lowered expectation. Specifically, we seek to show that even when expectation is confirmed, those who had set a low expectation to avoid failure actually end up feeling disappointed and less happy compared to those who did not.

### Experimental Design

We tested our hypothesis using a 2 (approach vs. avoidance motive) × 2 (information at feedback: performance-only vs. performance-and-goal) between-subjects design. One hundred and thirty-nine undergraduate and graduate business students at a large public university participated in the experiment for extra credit in an on-campus lab. Once seated, all participants were welcomed and provided information on the purpose and description of the study and time required (approximately 20 min). They were also informed that their responses will be unidentifiable and remain anonymous, and that participation was entirely voluntary. The students clicked “yes” to provide consent before proceeding and were debriefed of the purpose of the study upon completion. Ethics approval was not required as per the institution’s guidelines and national regulations; the study was exempted from the ethics review process by the Ethics Committee (Dr. Augustine Kposowa, Chair, and Dr. Rollanda O’Connor, Vice Chair, Human Research Review Board, Office of Research Integrity, University of California, Riverside).

#### Stimuli and Procedure

The experiment included two phases. In the first phase, participants were asked to complete two tasks framed either in an approach or an avoidance manner. The first task involved proofreading a short article in which the participants, in the approach condition, were instructed to “find the maximum number of misspelled words.” In the avoid condition, participants were instructed to “avoid missing any misspelled words” (Zhou and Pham, 2004). The second task was to solve a paper-and-pencil maze (Friedman and Forster, 2001) in which participants had to guide a cartoon mouse from the center of the maze to the exit. In the approach condition, a piece of cheese was depicted as sitting at the exit, whereas in the avoidance condition, a prowling owl was depicted as looking over the mouse from the opposite end of the exit. According to Friedman and Forster (2001), the cheese and the owl operationalize cognitive representation of “seeking reward” and “avoiding punishment,” respectively. It was predicted that, compared to those who were primed with an approach frame, participants primed with an avoidance frame would set more conservative return goals. It is noted that the manipulation used was originally designed for regulatory foci priming in which constructs promotion and prevention foci are close correlates of approach and avoidance motivations (Friedman and Förster, 2005, 2013).

In the second phase, participants completed the main experiment using an online financial investment interface. Performance is always at the level of the goal; that is, goals are met in all conditions. Participants were told that they would make investment decisions and receive feedback on their performance based on the actual performance of the stocks they picked. They were told:

“Imagine that you are living in a foreign country and need to invest your money. You have an investment budget of $5,400 and want to invest it in the stock market of this country. Given the market conditions in this country, at the end of a month, you can expect your portfolio to yield between 0 and 20% in return. As with any investment in a financial market, investing in stocks involves risk.”

On the next page, participants read, “You make investment decisions on the first of every month – that is, you trade on the 1st of each month. It is now the first day of March. What is the rate of return you would be satisfied with for this month?” Participants then picked a target level of return from the following possible target returns: 0, 2, 4, 6, …20%. Next, they responded to a manipulation check question to verify their awareness of the relative level of their expectation levels: “What is the level of expectation you have set for your portfolio’s performance?” (1-low expectation; 9-high expectation).

On the following page, participants constructed stock portfolios using an interactive interface. They were presented with a list of 20 fictitious stocks along with key information such as P/E ratio, price, ROE, debt-to-equity ratio, and EPS (quarter vs. year ago). The interactive interface simulated information layout of the E^∗^Trade website. The experimental program recorded the time participants spent on reviewing, selecting, and allocating the three stocks. Low- and high-goal setters did not differ in terms of the amount of time that they spent on the task [*M*_logoal_ = 7 min 48 s vs. *M*_higoal_ = 7 min 12 s; *F*(1,139) = 0.88, *n.s.*].

After a ten-minute filler task, participants were given feedback and received their stock portfolio returns on the subsequent screen. The returns matched their goals (goal+0.04%, the latter added to increase believability of the feedback). Participants were led to believe that their stock picks and allocations were used to calculate the actual returns using real data for that month for which they made their decision. For the “performance-only feedback” (default) condition, only the performance information was provided at feedback. Those in the “performance-and-goal at feedback” condition were told, “You had predicted that you would be satisfied with (actual goal) % for the past month. Your portfolio has resulted in a return of (actual goal plus 0.04%).”

Respondents then recorded their thoughts about their performance (“Please write down all thoughts that came to mind when you saw the performance level of your stock portfolio.”). They next rated their disappointment with the performance of their stocks on a 9-point scale. As a final separate study, respondents were asked to complete a set of “personality questionnaires,” which included a set of questions designed to measure their affect-management concern (“I chose a performance goal that would reduce my future disappointment”; “Choosing a low goal is better than high goal because it is helpful in dealing with anxiety”; “Setting a low goal is a good way to prepare for an uncertain outcome”; “I kept in mind that not meeting my goal would make me unhappy”; 9-point scale; 1 = not at all agree; 9 = definitely agree; *alpha* = 0.72). Affect-management concern measures were collected to test the role of an anticipatory negative affect in goal setting. Our prediction is that concern regarding a future affect, and the desire to avoid a negative affect, will mediate the predicted effect of avoidance prime on lowering goals. Finally, we collected involvement and expertise measures, which did not differ across conditions.

## Results

### Manipulation Check

To verify that those participants who managed their expectation by setting low goals were aware that they chose a low target (vs. high) we regressed the self-rated level of expectation item to the actual level of goal. As anticipated, the level of goals set and perceived level of these goals were strongly correlated (*r* = 0.36, *p* < 0.0001).

### Setting Goals Low to Avoid Negative Emotion

We predicted that individuals lowball their goals to avoid uncertainty and a negative affect from failure. As expected, participants under an avoidance frame (vs. approach frame) were found to set significantly lower goals (i.e., target rates of return) (β = -0.61, *p <* 0.0001). We then tested the mediating role of affect-management concern (combined measure of four question items; min = 1, max = 9) on level of goals set, using Hayes’ (2013) PROCESS model 4. A bootstrapping analysis (5,000 iterations) revealed that affect-management concern had a significant, indirect effect on the level of goal set (β = 0.43, 95% confidence interval [CI]: [0.15, 0.79]). This result suggested that goal setting under uncertainty could serve as an anticipatory coping mechanism to avoid a negative affect. Isolating the measure for salience of disappointment, the indirect effect of the desire to avoid disappointment on the goal level was stronger under avoidance motivation compared to approach motivation (β = 0.51, 95% confidence interval [CI]: [0.18, 0.89]).

Analyses were conducted using only the respondents in the approach frame group, who set their goals on the high end of the 0 to 20% return scale (≥10% median goal set) and those in the avoidance frame group, who set their goals on the low end of the scale (<10%; *N* = 124). Restricting the data was to ensure against concerns of self-selection, or third variable problem, due to chronic tendency such as general optimism and pessimism. The main question was whether this strategy of lowering goals actually helps one to avoid a negative emotion. In other words, does lowering one’s goal help in avoiding disappointment?

### Disappointment

**FIGURE 1:**
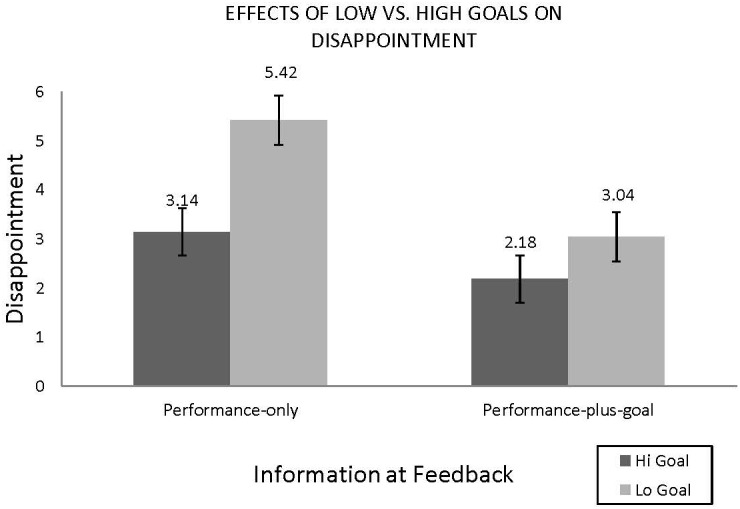
Study 1 results. *N* = 124, disappointment measured on 9-point scales (1 = not at all; 9 = extremely).

The hypothesis was tested using a regression analysis with “disappointment” as the dependent variable (DV) and the selected goal level (between 0 and 20% return, mean-centered) and feedback (performance-only vs. performance-and-goal) as the inDVs. The analysis revealed a significant main effect of goal level (goal β = -0.29, *p* < 0.0001) such that disappointment increased as the level of goal decreased. There was also a main effect of providing a goal at feedback such that reminding participants of their goal at feedback led to increased satisfaction (β = 0.80, *p* < 0.01). The interaction between the feedback variable (goal reminded or not) and the goal level was directionally consistent but not significant (*β* = -0.43, *p* = 0.13). More to our interest is whether those who lower their goals and actually achieve their goals are helping themselves to avoid the negative affect of disappointment. As illustrated in Figure 1, within the performance-only condition, those who achieve their low, safe goals report greater disappointment than those who set and achieve a higher goal. Follow-up contrasts confirmed that participants were more disappointed when they had set a low-vs.-high goal (based on median of 10%) in the performance-only feedback condition (*M*logoal = 5.42 vs. *M*higoal = 3.14), [*F*(1,120) = 27.17, *p* < 0.0001]. Providing the initial goal along with the performance at the time of feedback eliminated the effect of setting low-vs.-high goals (*M*logoal = 3.04 vs. *M*higoal = 2.18), (*F* = 3.45, *p* = 0.09).

### Discussions and Limitations

The higher disappointment reported by the low-expectation individuals suggests that setting low expectations is not conducive to averting disappointment despite having such expectations confirmed. Those who manage their expectations and have them confirmed were more disappointed compared to those who did not. The interaction of providing goals at the feedback evaluation suggests that the portfolio-performance comparison was not to the initially set goals. This result is consistent with Cho and Johar (2011) finding on shifting reference standards whereby people were observed to invoke superior, alternative reference standards when they evaluate their performance. Results from experiment 1 suggest that the desire to manage a future affect by lowering a target goal is not conducive to success feedback. This finding suggests that in fact, trying to manage future success undermines the self-regulatory feedback loop by instigating perceptions of failure. Awareness of the initial goal at feedback remedies this effect. It is interesting to note that all participants reported feeling disappointed with the outcome, although in different degrees. This finding is consistent with extant research on counterfactual thinking and the general tendency to upwardly compare to unattained, alternative outcomes (see, for example, Roese, 1997).

A limitation of this study was that the level of goal and the performance outcome were the same. Although this was by design, it lends to the concern that performance outcome that varied may drive the effect. Therefore, as the purpose was to examine the effect of managing expectations (not outcome) on emotional reactions to a confirmed expectancy, holding the outcome constant would lend additional strength to the findings. This limitation notwithstanding, we note that it does not take away from the core premise – that is, all participants in the study were choosing the target (expectations) that they considered would be satisfactory. The use of the stock-picking task, while interactive and self-involving, could be subject to the concern that it is artificial and unfamiliar. Replication of the results using real monetary consequences would enhance this concern. Further, replicating the findings using other domains of self-relevance would enhance generalizability of this study’s findings. While different in scope, we explore more familiar and self-involving domains in our next study.

## Study 2

Study 1 demonstrated that setting goals low might be counterproductive in avoiding disappointment even if the goals are met. This study examines the possibility that greater awareness of the difficulty of forecasting future emotion may reduce the lowballing tendency among those with a chronic tendency to set low goals. Whereas the preceding study primed participants to set different goal levels, this study examines chronic tendencies to set low or high goals, with the level of goal set as the main DV. One such chronic tendency, or trait difference that may dictate whether goals are chronically set low or high is regulatory focus (Shah et al., 1998). According to Shah et al. (1998), people can construe aspirational standards as minimal goals they must attain or as maximal goals they hope to attain. Minimal goals differentiate negative from non-negative events, whereas maximal goals differentiate positive from non-positive events (Brendl and Higgins, 1996; Idson et al., 2000). Freitas et al. (2002) have demonstrated that a promotion focus tends to foster concerns with maximal goals while a prevention focus fosters concerns with minimal goals. Because goals within a promotion focus are seen as opportunities to try for optimal outcomes – whereas goals within a prevention focus are perceived as minimal requirements – it could be argued that this dispositional tendency in goal perception influences the actual level of aspiration one sets for oneself. Specifically, an individual with a prevention focus is likely to set low goals because he is concerned with avoiding failure and achieving the minimum is perceived to fit this goal. In contrast, a promotion-focused individual is likely to set higher goals because he is less concerned with avoiding failure and the accompanying disappointment.

Can this tendency to set low goals be mitigated? One method of intervention would be to increase awareness regarding the fallacy that one can avert disappointment by managing expectations. If individuals are made aware that anticipated affect is often misleading and inaccurate, the tendency to set preemptively low goals in anticipation of a possible negative affect could be prevented. Research on context effects and bias correction has shown that, when people become aware of their reactions as being due to primes rather than the target, they attempt to “correct” their reactions by consciously “resetting” and adjusting their judgments (Martin, 1986; Schwarz and Bless, 1992; Wegener and Petty, 1997). In other words, when made aware of a potential bias, individuals would attempt to correct for this bias. This motivation to correct, we reason, should also operate when an individual is made aware of the tendency to overpredict a potential negative affect and the worst outcome.

In this study, we test to see whether making participants aware of the inaccuracy of the lay theory (tendency to overpredict negative emotion and the belief that lowering goals and expectations can help to avoid it) can trigger a corrective process, whereby the lowballing of goal is mitigated. We expect that those who are aware of the inaccuracy of their predicted affect will effectively set higher goals, particularly those who are chronically likely to lower goals as measured by their regulatory orientation.

### Experimental Design

The study used a 2 (prime: awareness vs. no awareness) × 2 (chronic regulatory focus: promotion vs. prevention) between-subjects design with the level of goal as the main DV. One hundred and thirty participants participated as part of extra credit for a class. As with study 1, all participants were informed of the confidentiality and unidentifiability of their responses and that their participation was entirely voluntary. They were told that they would perform several unrelated tasks consisting of a reading comprehension task, a stock-picking study, and a lifestyle questionnaire. The students clicked “yes” to provide consent before proceeding and were debriefed of the purpose of the study upon completion. As with study 1, this study was approved by the university’s Human Research Review Board. Ethics approval was not required as per the institution’s guidelines and national regulations; the study was exempted from the ethics review process by the Ethics Committee (Dr. Augustine Kposowa, Chair, and Dr. Rollanda O’Connor, Vice Chair, Human Research Review Board, Office of Research Integrity, University of California, Riverside).

#### Stimuli and Procedure

In the first study, presented as a comprehension study given by the English department, respondents in the “awareness” prime condition read a newspaper article describing research by Gilbert and Wilson (2000, c.f. Gertner, 2003) on the unreliability of forecasted feelings. Those in the “no awareness” prime condition read an article about strategies to avoid disappointment to succeed in life. Participants were asked to rate their agreements on five question items using a 7-point scale (1 = strongly disagree and 7 = strongly agree): “This article was convincing”; “It is wise to avoid disappointment in navigating life’s challenges”; “Managing one’s expectation is a good strategy to cope with life’s uncertainties and disappointments”; “To be happy, it is important not to think so much about possible failures and disappointments”; “It is important to know that what people expect will be disappointing or painful will not be so bad.”

In the next ostensibly unrelated study, the scenario used was similar to that in study 1, except for returns that ranged from negative to positive, (“Given the market condition in this country, at the end of a month, you can expect your portfolio to yield between -20 and 20% in return.”). Participants worked on a computer and were led to believe that the study would entail an actual stock-picking task after which they would be provided how well their chosen stocks performed (identical to study 1); but because we are interested in the target level chosen as the main DV, the study would stop when they chose a level of return. Participants read the stock-picking scenario and chose from a set of 21 possible levels of returns (0, 1, 2, 3, …, 20%). In addition, in the next (unrelated) “lifestyle and value survey” that followed, participants were asked to state their target in various life domains, namely saving money, exercising, losing weight, and any other goal they may have previously set, and whether these goals were easy or difficult. These questions were expected to serve as replicates of the predicted interaction effect of regulatory foci and awareness of inaccuracies of the lay theory on managing goal levels.

In the final “unrelated study,” respondents answered the 11-item Regulatory Focus Questionnaire (Higgins et al., 2001) where they rated their history of promotion and prevention success and failure on 5-point scales (1-never or seldom; 5-very often). Participants were then debriefed and told that the study was concerned with goal-setting rather than financial decision-making. We expected that chronic orientations on the promotion and prevention dimensions would correlate with the level of goals set such that a prevention focus would lower the goal level. Specifically, it is predicted that prevention individuals will set lower goals than promotion individuals, and that within the awareness condition, this tendency will not be observed whereby prevention individuals will set their goals at a similar level as compared to promotion individuals.

## Results

### Manipulation Check

An ANOVA was run on the measures of agreement with the convincingness of the article that was read. Nine respondents who rated the article as completely unconvincing were dropped from the study (seven from no awareness and two from awareness conditions), as were six participants whose numerical responses were identical throughout the questionnaires, resulting in a sample of 115 participants. Next, ANOVA was run on measures of agreement with the effectiveness of the goal-lowering strategy (“To be happy and successful, it is important to avoid disappointments in life,” “Managing one’s expectation is a good strategy to cope with life’s uncertainties and disappointments.”) and on two measures of agreement with the tendency to exaggerate future negative affect (“To be happy, it is important not to think so much about failure and disappointment”; “To be happy, it is important to know that what we think will be disappointing or painful will not be so bad.”). The two pairs of measures were highly correlated and combined for analysis (*r* = 0.68, *p* < 0.0001; *r* = 31, *p* < 0.001). As expected, those in the no-awareness condition were significantly greater in agreement (*M* = 5.16) on the effectiveness of the lowballing strategy compared with the awareness condition [*M* = 4.27; *F*(1,113) = 12.26, *p* < 0.001]. The awareness condition group was also better informed regarding the inaccuracies of the lay theory in avoiding a future negative affect compared to the no-awareness condition (*M* = 5.69 vs. 5.03, *p* < 0.005).

### Goal Setting

**FIGURE 2:**
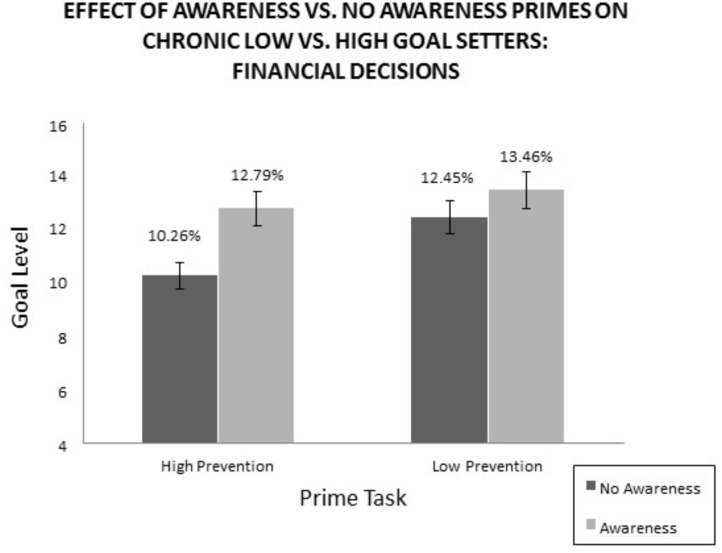
Study 2 results. *N* = 115, goal measured in percent rate of possible returns (goal choices provided: 0–20%).

Level of goals chosen, the main DV of interest, was analyzed first by regressing goal level to prime type (awareness vs. no awareness) and Regulatory Focus (RF) Questionnaire scores (Higgins et al., 1997). Consistent with the RF theory which holds that an individual can be high or low in prevention and promotion foci, that is, that the motivational orientations are orthogonal, the prevention and promotion scores did not correlate (Pearson *r* = 0.09). Regression analyses for promotion and prevention foci as predictor, goal level chosen as DV, and awareness treatment vs. control treatment as moderator were conducted. As predicted, the chronically prevention-focused individuals were more likely to set a low goal (standardized β = -0.21, *p* < 0.05), whereas promotion focus had no significant effect (standardized β = -0.07, *n.s.*). Given that promotion and prevention foci are orthogonal, we proceeded to focus on individual differences in prevention orientation (high vs. low) as the predictor variable in our analysis. Moderator and interaction between predictor and moderator were not significant. More to our interest is whether the chronic tendency to lower the level of goal by the high prevention individuals can be intervened. Therefore, an ANOVA was run for the 2 (Prevention: High vs. Low) × 2 (prime type: awareness vs. no awareness) study, with the prevention measure separated into high or low at the median (high if >19). There was a marginally significant effect of the prevention focus on the level of goal [*F*(1,111) = 3.5*, p* = 0.07] such that high-prevention respondents were setting directionally lower goal levels compared to the low-prevention respondents. More to our interest is whether the high-prevention individuals, who are presumably the chronic low goal setters and prone to managing expectations, can “correct” this tendency when made aware of the tendency to over-anticipate negative emotion. In other words, we tested whether after being made aware of the fallibility of predicting future emotion, the lowering of goals can be prevented. Results supported this prediction (see Figure 2). Within the high-prevention group, those who read the awareness treatment set their goals significantly higher (*M* = 13.12%) than those in the no- awareness group (*M* = 11.00%), [*F*(1,111) = 4.11, *p* < 0.05]. In comparison, those in the low-prevention group did not differ significantly in their level of goals (*M*_disapp_ = 12.25%, *M*_correct_ = 13.26%), (*F* < 1). Notably, the hi-prevention group under the awareness prime set goals that were not significantly different from the promotion group, suggesting that the dispositional tendency to lowball on goals (together with the negative emotion of disappointment) may be “corrected” by increasing awareness of the inaccuracies in forecasting one’s future affect.

Following the stock-picking scenario study, participants also completed the “General Lifestyle Survey,” in which they were asked to rate their goals in various domains, such as saving money, going to the gym, and losing weight (Studies 2a, 2b, and 2c, Table 1). It is noted that this study was conducted in mid-January and February, and some of the questions were designed to correspond to seasonal concerns. The participants were asked to set their goals for three other goal-setting domains: Exercise, (“Exercising and going to the gym is an important part of staying healthy. How often do you plan to go to the gym this year?”; 1-never, 2-once a month, 3-twice a month, 4-three times a month, 5-four times a month, 6-once a week, 7-twice a week, 8-three times a week, 9-four times a week or more); saving (“People often set savings goals to curb their spending. What is the amount of savings you would strive for this year?”; less than 5%, 5%, 10%, 15%, 20%, 25%, 30%, 35%, 40% or more); and losing weight (“Losing weight is an important part of maintaining health. What is your goal for losing and maintaining your weight? In other words, how many pounds do you plan to lose this winter season?”; open-ended response). Participants were then asked to rate the perceived difficulty of the goal they indicated using a 9-point scale (For you, this goal is…: 1-very easy; 3-somewhat easy; 5-neither difficult nor easy; 7-somewhat difficulty; 9-very difficult). We used the perceived difficulty of the goal as the main measure of goal level chosen.

**Table 1:** Studies 2 results.

Motivation	Awareness condition	Study 2: return goal level (*N*=113)	Study 2a: savings goal (*N*=113; 9-very difficult; 1-very easy)	Study 2b: exercise goal (*N*=113; 9-very difficult; 1-very easy)	Study 2c: weight loss goal (*N*=113; 9-very difficult; 1-very easy)
Promotion	Awareness	13.46%	5.46	5.21	5.68
	No awareness	12.45%	6.07	5.77	5.57
Prevention	Awareness	12.79%	5.45	5.58	5.38
	No awareness	10.26%	4.56	3.07	3.89


Interestingly, the patterns of results were similarly in support of the finding that the chronically low-goal setting (high prevention-focused) individuals who read the one-page description of the affective forecasting research (awareness prime) chose goals that were more difficult (compared to low prevention-focused individuals). For the exercise goal, high-prevention individuals who read the awareness treatment set their goals significantly higher in rated difficulty (*M* = 5.58) than under the no-awareness treatment (*M* = 3.07), [*F*(1,111) = 18.40, *p* < 0.0001], while there was no difference for the low-prevention individuals (5.21 vs. 5.77, *n.s.*) (Appendix 1, in Supplementary Material). For savings, for the high-prevention individuals, those in the awareness treatment condition set their goals directionally higher in difficulty (*M* = 5.46) than under the no-awareness treatment (*M* = 4.56), [*F*(1,111) = 2.94, *p* = 0.08]; there was no difference for the low-prevention individuals (5.46 vs. 6.06*, n.s*.) (Appendix 2, in Supplementary Material). Similarly, for losing weight, the pattern was significant and in a similar direction. High-prevention individuals in the awareness condition were setting significantly more difficult goals than high-prevention individuals in the no-awareness treatment [5.38 vs. 3.39; *F*(1,111) = 5.71, *p* < 0.05], while no such effects were observed for the low-prevention individuals (5.58 vs. 5.68, *n.s.*) (Appendix 3, in Supplementary Material).

## Discussion

The purpose of Study 2 was fairly straightforward. That is, given that concern with managing future disappointment and negative emotion leads to a counterproductive tendency to lower one’s goal, could this tendency be prevented by making better information available about the misguided assumptions behind such a tendency? To verify the premise that a salient desire to avoid disappointment can motivate one to “manage expectations” and set a low goal, we used chronic differences in concerns of avoiding negative outcomes using the motivational construct of a prevention focus. We tested our hypothesis on individuals with different regulatory orientation as a proxy for chronic low-goal setting tendency and found that the chronic tendency may be “corrected” when individuals are made aware that lowering goals is not always helpful in maximizing happiness. While financial decision-making domain was chosen for the parsimonious operationalization and replication of previous research (Cho and Johar, 2011), we test and find consistent results using three additional domains of goal setting. The simple replication across the four domains provides additional validation to the generalizability of awareness as a way to mitigate the goal setting behavior.

We manipulated motivational states to induce greater sensitivity to avoiding negative outcome (study 1) as well as measured trait differences (study 2). We tested the efficacy of this implicit strategy of goal lowering and expectations in averting disappointment (study 1) and more importantly, to explore ways to intervene against the potentially negative impact of such a strategy. Our findings suggest that an intervention of a reminder of the initial goal (study 1) or making individuals aware of the potential inaccuracies of trying to manage their future affect (study 2) may help to alleviate the likely negative emotional impact.

Study 1 demonstrated that helping consumers retain their original goals and perspectives might counter the potential impact of upward comparison and the negative emotion that accompanies this process. This study tested more directly whether adverse emotional consequence of lowballing one’s expectation could be countered. It was demonstrated that better informing the consumers might be effective in correcting the misguided tendency to lower one’s expectations. The results suggest that better informing individuals of the difficulties in accurately forecasting their emotional state can effectively deactivate resorting to lowering one’s goals and expectations.

## General Discussion

This research investigated the equivocal claim that managing expectations and setting low goals can help to prepare for a potentially negative outcome. While various streams of research including bracing (Shepperd et al., 1996) and the expectancy-disconfirmation model (Oliver, 1980; Van Dijk et al., 2003) have lent support to this lay strategy of “managing expectations,” more recent research has offered boundary conditions under which such a strategy may backfire (Ordonez et al., 2009; Townsend and Liu, 2012). Our findings are compatible with previous findings that show that, when goals and expectations are motivated and set low, it may not be stable enough to serve as a reference standard when one’s performance outcome is revealed and assessed. Given the instability of reference standards, and the negative emotional consequences, the two studies tested ways to intervene against the counterproductive strategy of setting low expectations. More generally, making individuals aware of the likely inaccuracies in predicting what would be satisfactory in the future, namely, the demonstrated tendency to forecast one’s affect inaccurately (Gilbert and Wilson, 2000; Wilson and Gilbert, 2003), may offer a simple remedy for the negative impact of “managing expectations.” An interesting avenue for future research would be whether the negative consequence of managing expectation may operate on an organizational level (Locke et al., 1990). Existing research on goals in the workplace suggests that setting goals that are too high can motivate unethical behavior (e.g., Schweitzer et al., 2004). By the same token, it would be worthwhile to examine the possibility that setting too manageable an expectation may be counterproductive in optimally motivating employees. The findings from this study also illuminate the implications of earnings management for stakeholder groups, as well as the capital market (Hirst et al., 2008).

## Author Contributions

CC initiated the theoretical motivation for the paper, while TC and CC collaborated on the study design and data collection and analyses. CC wrote the first draft and went through multiple iterations with TC.

## Conflict of Interest Statement

The authors declare that the research was conducted in the absence of any commercial or financial relationships that could be construed as a potential conflict of interest.

## References

[B1] Aspinwall L. G. (2005). The psychology of future-oriented thinking: from achievement to proactive coping, adaptation, and aging. *Motiv. Emot.* 29 203–235. 10.1007/s11031-006-9013-1

[B2] Bandura A. (1982). Self-efficacy mechanism in human agency. *Am. Psychol.* 37 122–147. 10.1037/0003-066X.37.2.122

[B3] Brendl C. M. Higgins E. T. (1996). “Principles of judging valence: what makes events positive or negative?,” in *Advances in Experimental Social Psychology* Vol. 28 ed. Zanna M. P. (San Diego, CA: Academic Press) 95–160.

[B4] Carroll P. Sweeny K. Shepperd J. A. (2006). Forsaking optimism. *Rev. Gen. Psychol.* 10 56–73. 10.1037/1089-2680.10.1.56 12051481

[B5] Carver C. S. Scheier M. F. (1990). “Principles of self-regulation: action and emotion,” in *Handbook of Motivation and Cognition: Foundations of Social Behavior* Vol. 2 eds Sorrentino R. M. Higgins E. T. (New York, NY: Guilford Press), 3–52.

[B6] Carver C. S. Scheier M. F. (1998). *On the Self-Regulation of Behavior.* New York, NY: Cambridge University Press 10.1017/CBO9781139174794

[B7] Cho C. K. Johar G. V. (2011). Attaining satisfaction. *J. Consum. Res.* 38 622–631. 10.1086/660115

[B8] Forster J. Higgins E. T. Idson L. C. (1998). Approach and avoidance strength during goal attainment: regulatory focus and the “goal looms larger” effect. *J. Pers. Soc. Psychol.* 75 1115–1131. 10.1037/0022-3514.75.5.1115 9866180

[B9] Freitas A. L. Liberman N. Higgins E. T. (2002). Regulatory fit and resisting temptation during goal pursuit. *J. Exp. Soc. Psychol.* 38 291–298. 10.1006/jesp.2001.1504

[B10] Friedman R. Forster J. (2001). The effects of promotion and prevention cues on creativity”. *J. Pers. Soc. Psychol.* 81 1001–1013. 10.1037/0022-3514.81.6.1001 11761303

[B11] Friedman R. S. Förster J. (2005). Effects of motivational cues on perceptual asymmetry: implications for creativity and analytical problem solving. *J. Pers. Soc. Psychol.* 88 263–275. 10.1037/0022-3514.88.2.263 15841858

[B12] Friedman R. S. Förster J. (2013). “Activaton and measurement of motivational states,” in *Handbook of Approach and Avoidance Motivation* ed. Elliot A. (New York, NY: Psychology Press).

[B13] Gertner J. (2003). The futile pursuit of happiness. *The New York Times* 7th September.

[B14] Gilbert D. T. Wilson T. D. (2000). “Miswanting: some problems in the forecasting of future emotional states,” in *Thinking and Feeling: The Role of Affect in Social Cognition* ed. Forgas J. (Cambridge: Cambridge University Press) 178–197.

[B15] Hayes A. F. (2013). *Methodology in the Social Sciences. Introduction to Mediation, Moderation, and Conditional Process Analysis: A Regression-Based Approach.* New York, NY: Guilford Press.

[B16] Higgins E. T. (1987). Self-discrepancy: a theory relating self and affect. *Psychol. Rev.* 94 319–340. 10.1037/0033-295X.94.3.319 3615707

[B17] Higgins E. T. (1996). The self digest. self-knowledge serving self-regulatory functions. *J. Pers. Soc. Psychol.* 71 1062–1083. 10.1037/0022-3514.71.6.1062 8979379

[B18] Higgins E. T. (1997). Beyond pleasure and pain. *Am. Psychol.* 52 1280–1300. 10.1037/0003-066X.52.12.1280 9414606

[B19] Higgins E. T. Friedman R. S. Harlow R. E. Idson L. C. Ayduk O. N. Taylor A. (2001). Achievement orientations from subjective histories of success: promotion pride versus prevention pride. *Eur. J. Soc. Psychol.* 31 3–23. 10.1002/ejsp.27

[B20] Higgins E. T. Shah J. Friedman R. (1997). Emotional responses to goal attainment: strength of regulatory focus as moderator. *J. Pers. Soc. Psychol.* 72 515–525. 10.1037/0022-3514.72.3.515 9120782

[B21] Hirst D. E. Koonce L. Venkataraman S. (2008). Management earnings forecasts: a review and framework. *Account. Horiz.* 22 315–338. 10.2308/acch.2008.22.3.315

[B22] Idson L. C. Liberman N. Higgins E. T. (2000). Distinguishing gains from non-losses and losses from non-gains: a regulatory focus perspective on hedonic intensity. *J. Exp. Soc. Psychol.* 36 252–274. 10.1006/jesp.1999.1402

[B23] Jain S. P. Agrawal N. Maheshwaran D. (2006). When more may be less: the impact of regulatory focus on responses to different comparative frames. *J. Consum. Res.* 33 91–98. 10.1086/504139

[B24] Larrick R. P. Heath C. Wu G. (2009). Goal-Induced risk taking in negotiation and decision making. *Soc. Cogn.* 27 342–364. 10.1521/soco.2009.27.3.342

[B25] Locke E. A. Latham G. P. Smith K. J. Wood R. E. (1990). *A Theory of Goal Setting and Task Performance.* Upper Saddle River, NJ: Prentice Hall College Division, 544.

[B26] Martin L. (1986). Set/reset: use and disuse of concepts in impression formation. *J. Pers. Soc. Psychol.* 51 493–504. 10.1037/0022-3514.51.3.493 3761145

[B27] McClelland D. C. (1953). *The Achievement Motive.* Eastford, CT: Martino Fine Books 10.1037/11144-000

[B28] Monga A. Houston M. J. (2006). Fading optimism in products: temporal changes in expectations about performance. *J. Mark. Res.* 18 654–663. 10.1509/jmkr.43.4.654

[B29] Oliver R. L. (1980) A cognitive model of the antecedents and consequences of satisfaction decisions. *J. Mark. Res.* 17 460–469.

[B30] Ordonez L. Schweitzer M. E. Galinsky A. D. Bazerman M. H. (2009). Goals gone wild: the systematic side effects of overscribing goal setting. *Acad. Manag. Perspect.* 23 6–16. 10.5465/amp.2009.37007999

[B31] Roese N. J. (1997). Counterfactual thinking. *Psychol. Bull.* 121 133–148. 10.1037/0033-2909.121.1.133 9000895

[B32] Schwarz N. Bless H. (1992). “Constructing reality and its alternatives: an inclusion/exclusion model of assimilation and contrast effects in social judgment,” in *The Construction of Social Judgments*, eds Martin L. L. Tesser A. (Hillsdale, NJ: Erlbaum), 217–245.

[B33] Schweitzer M. E. Ordóñez L. Douma B. (2004). Goal setting as a motivator of unethical behavior. *Acad. Manag. J.* 47 422–432.

[B34] Shah J. Higgins E. T. Friedman R. S. (1998). Performance incentives and means: how regulatory focus influences goal attainment. *J. Pers. Soc. Pers.* 74 285–293. 10.1037/0022-3514.74.2.285 9491583

[B35] Shepperd J. A. Ouellette J. A. Fernandez J. K. (1996). Abandoning unrealistic optimism: performance estimates and the temporal proximity of self-relevant feedback. *J. Pers. Soc. Psychol.* 70 844–855. 10.1037/0022-3514.70.4.844

[B36] Townsend C. Liu W. (2012). Is planning good for you? The differential impact of planning on self-regulation. *J. Consum. Res.* 39 688–703. 10.1086/665053

[B37] Van Dijk W. W. Zeelenberg M. van der Pligt J. (2003). Blessed are those who expect nothing: lowering expectations as a way of avoiding disappointment. *J. Econ. Psychol.* 24 505–516. 10.1016/S0167-4870(02)00211-8

[B38] Wegener D. T. Petty R. E. (1997). “The flexible correction model: the role of naïve theories of bias in bias correction,” in *Advances in Experimental Social Psychology* Vol. 35 ed. Zanna M. (New York, NY: Elsevier) 142–208.

[B39] Wilson T. D. Gilbert D. (2003). “Affective forecasting,” in *Advances in Experimental Social Psychology* Vol. 35 ed. Zanna M. (New York, NY: Elsevier) 345–411.

[B40] Zhou R. Pham M. T. (2004). Promotion and prevention across mental accounts: when financial products dictate consumers’ investment goals. *J. Consum. Res.* 31 125–135. 10.1086/383429

