# Supplementary figures and images for: On Averting Negative Emotion: Remedying the Impact of Shifting Expectations
Source: Front Psychol. 2018 Nov 20;9:2121. doi: 10.3389/fpsyg.2018.02121 (PMC6256416; doi:10.3389/fpsyg.2018.02121)

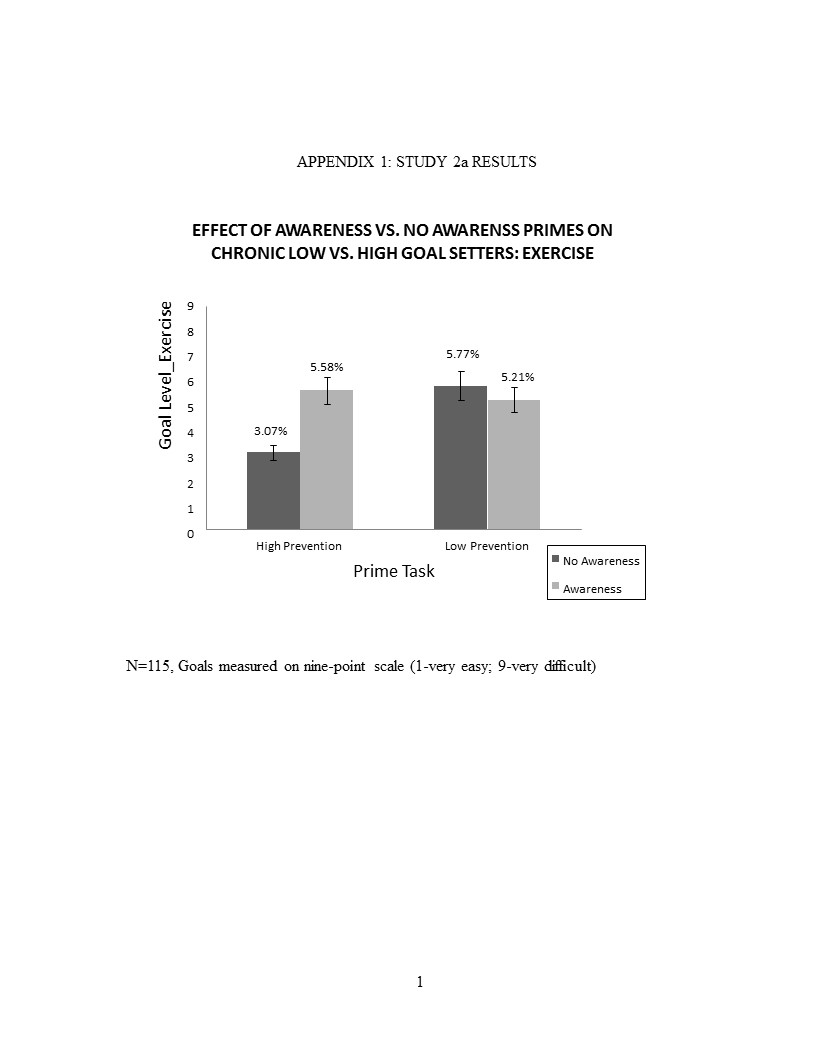

Supplement: Supplementary file 1 [file Image_1.jpeg]

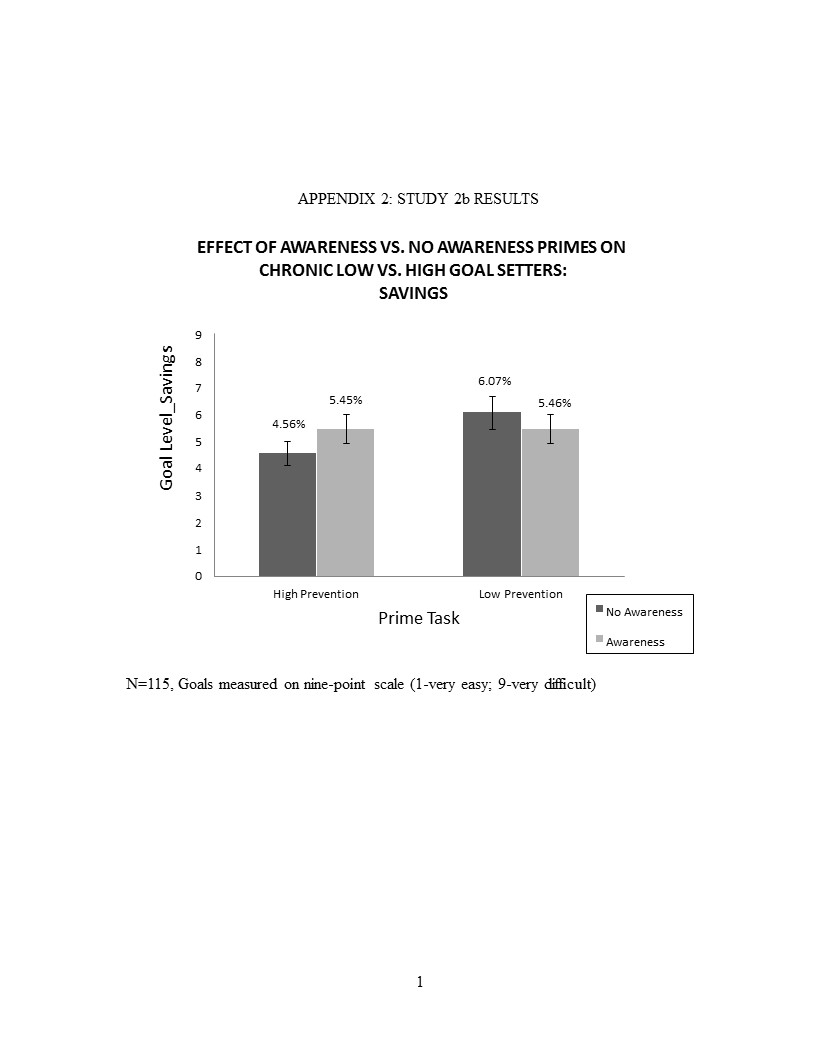

Supplement: Supplementary file 2 [file Image_2.jpeg]

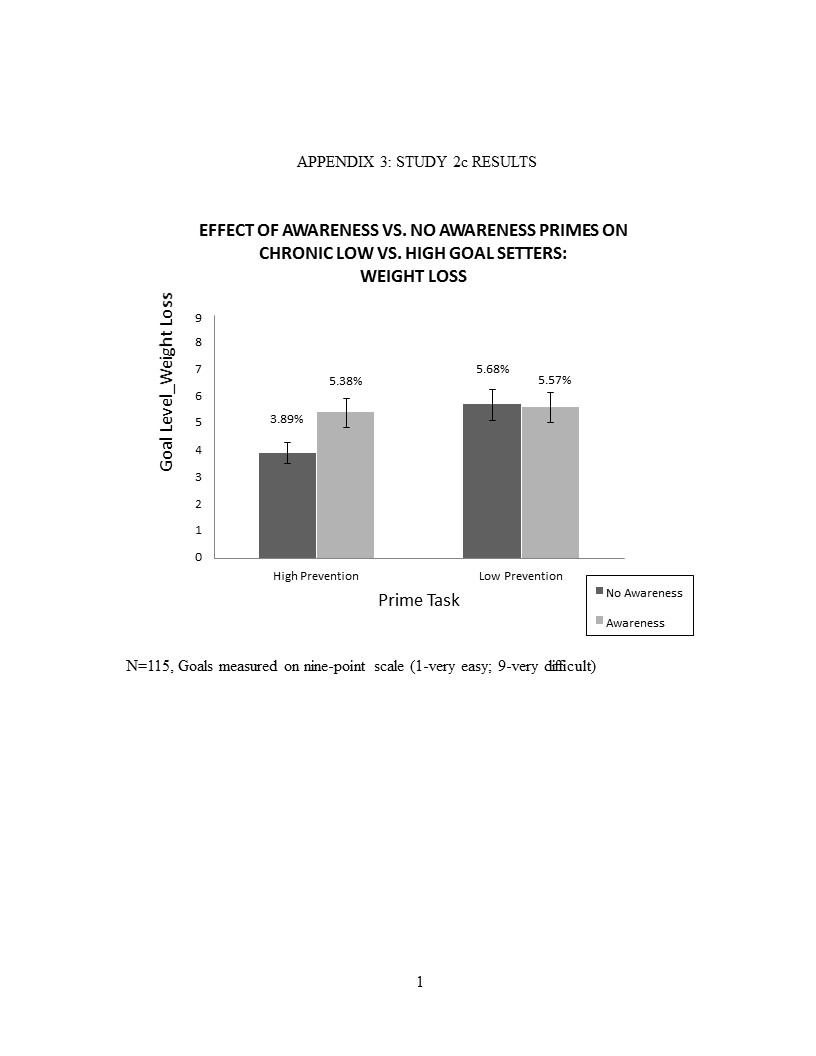

Supplement: Supplementary file 3 [file Image_3.jpeg]
